# Skin Tumors Rb(eing) Uncovered

**DOI:** 10.3389/fonc.2013.00307

**Published:** 2013-12-17

**Authors:** Clotilde Costa, Jesús M. Paramio, Mirentxu Santos

**Affiliations:** ^1^Molecular Oncology Unit, Department of Basic Research, Centro de Investigaciones Energéticas Medioambientales y Teconológicas (ed70A), Madrid, Spain

**Keywords:** pRb, p107, p130, epidermis, E2F, p53, SCC, transgenic mice

## Abstract

The *Rb1* gene was the first *bona fide* tumor suppressor identified and cloned more than 25 years ago. Since then, a plethora of studies have revealed the functions of pRb and the existence of a sophisticated and strictly regulated pathway that modulates such functional roles. An emerging paradox affecting *Rb1* in cancer connects the relatively low number of mutations affecting *Rb1* gene in specific human tumors, compared with the widely functional inactivation of pRb in most, if not in all, human cancers. The existence of a retinoblastoma family of proteins pRb, p107, and p130 and their potential unique and overlapping functions as master regulators of cell cycle progression and transcriptional modulation by similar processes, may provide potential clues to explain such conundrum. Here, we will review the development of different genetically engineered mouse models, in particular those affecting stratified epithelia, and how they have offered new avenues to understand the roles of the *Rb* family members and their targets in the context of tumor development and progression.

## Genetically Engineered Mouse Models

Mouse models are essential tools to analyze the molecular mechanisms underlying any physiological event that take place in the organism. They provide information about gene function, allow the analysis of specific genetic changes and, under appropriate circumstances, might mimic human diseases. This characteristic enables the close analysis of pathology and the identification and validation of candidate therapeutic targets.

Several approaches are used to generate genetically engineered mouse models (GEMMs). The traditional methodology, based on the elimination of the gene of interest in the whole animal (knockout mice) (1), allows the functional characterization of the gene product during the organism development. However, this may result in embryonic lethality, precluding the study in adult animal tissues. This problem can be circumvented by the use of conditional transgenic gene knockout (e.g., involving cre-loxP system), which allows studies of gene function in specific cell and tissue types, including adult ones (2). Additionally, both experimental systems also provide valuable information about potential compensatory mechanisms, since the possible functions of the specific ablated gene can be carried out by other genes. Such compensation could occur directly, if “compensatory” genes are up-regulated or down-regulated as a direct result of the loss of the gene under study. The existence of such overlapping roles may also help to determine possible cooperative events in disease onset or progression. The Rb and E2F families represent a paradigm of direct compensatory mechanisms revealed using conventional and conditional knockouts (3).

The conditional knockout mice are engineered in such way that genes can be inactivated in a tissue-specific manner. This approach requires the use of specific gene promoters, which in some circumstances may represent a problematic issue due to ectopic expression or poorly characterized control elements. Nonetheless, these GEMMs enable much more sophisticated pathology modeling, as they provide essential information of the potential role of a particular gene or group of genes in a determined cell type. This is of a particular relevance in cancer research where it is possible to determine whether the loss of a particular gene is involved in tumor susceptibility, initiation, or progression to malignancy. On the other hand, in sporadic cancer, initiating mutations probably occur in a unique or very few cells in a certain tissue. These initiated cells acquire proliferative or pro-survival advantages through subsequent genetic alterations and by a cross-talk with the microenvironment lead to tumor development and progression. Accordingly, most of the conventional or tissue-specific mouse models reproduce familial forms of cancer but not sporadic tumors, as mutations are present in every cell of the body or in vast majority of cells in a certain tissue (3). An interesting progress in the field was the development of a ligand-dependent Cre recombinase that can be activated by tamoxifen administration (4). These inducible mouse models, besides allowing the space-temporal control of recombination, also provide a better suited model of human sporadic cancer, as the recombination can be only limited to few cells in a given tissue.

## Skin as a Model System

Skin is an essential organ that forms a protective barrier against the environment. This function is primarily exerted by the epidermis, a stratified epithelium in the outermost layer of the skin, and relies on a finely tuned process of coupled differentiation and proliferation. Both processes are compartmentalized in this tissue, and can be characterized by the sequential expression of highly specific markers. The proliferative cells are confined to a single cell basal layer and the non-proliferative differentiating cells are located in the suprabasal layers. This represents one of the main characteristics that make epidermis an ideal model system. In particular, the expression of highly specific proteins in these compartments has allowed the characterization of specific promoters for different epidermal layers. In this context, it is remarkable the predominant use of the basal cell specific keratin K5 (5–7) and keratin K14 promoters (8), including the inducible specific forms (9).

Another attractive biologic characteristic of the epidermis resides in the cell replenishment and renewal processes occurring throughout the individual entire life. Terminally differentiated or damaged epidermal cells are shed from the skin, requiring a permanent tissue renewal fulfilled by epidermal stem cells. In mouse epidermis, these stem cells lie mostly in the hair follicle bulge, display a low proliferative rate and upon specific stress conditions (i.e., wound healing) can give rise to all the epidermal cell subtypes (10–12). Importantly, the hair follicle stem cells are also responsible for the hair cycling, which is coordinated by extremely relevant signaling pathways such as Wnt, BMP, Notch, etc. and they are considered the cells of origin in non-melanoma skin cancer (13–17). Epidermal stem cells can be isolated and characterized using different cell surface markers (18, 19) and they also express K5 and K14 genes. Accordingly, the use of the K5 and K14 control elements may also cause genetic alterations in these long-lived cells.

Besides the above commented attributes, epidermis is also perfectly suited to allow *in vitro* studies. Keratinocytes can be obtained and cultured *in vitro* as monolayer cell cultures. In these, differentiation can be achieved in response to external signals in a manner resembling that observed *in vivo* (20, 21). In humans, cultured keratinocytes, which can also be genetically modified, can be used to engineer skin equivalents (22–24), which can be grafted onto different receptors allowing the evolution of the skin transplant. In mice, skin transplants can avoid specifically complex technical approaches associated with compromised viability (25).

Finally, for the cancer field, the two-stage mouse skin carcinogenesis is perhaps the best characterized experimental carcinogenesis protocol and represents a suitable model for the understanding the multistage nature of tumorigenesis (26). This approach, besides allowing to define the relevance of multiple oncogenic and/or tumor suppressor activities, has also led to the establishment of fundamental molecular aspects of cancer, and has contributed in an essential manner to an ideal conceptual framework to understand many molecular aspects of tumorigenesis such as tumor angiogenesis, epithelial-mesenchymal transition and metastasis, and the role of adult stem cells (27).

## Retinoblastoma Family Genes

The retinoblastoma gene (*Rb1*), localized in chromosome 13q14.2, was the first tumor suppressor identified more than 25 years ago (28). Years later, other similar genes were discovered encompassing *Rb* family. They are the retinoblastoma-like 1 (*Rbl1*) and retinoblastoma-like 2 (*Rbl-2*) genes, localized in 20q11.2 and 16q12.2, and encoding p107 and p130 proteins, respectively (29–31). pRb, p107, and p130 share structural homology. These proteins are defined by a conserved pocket domain which serves as a binding site and provides the family name “pocket proteins.” The pocket domain consists of A and B domains separated by a spacer region, and is a region where viral oncoproteins bind (Ad-E1A, SV40 LT-antigen, or HPV-E7) (32, 33). Rb family proteins interact with proteins containing a LeuXCysXGlu (LXCXE) motif, found in several viral transforming proteins, such as HPV E7, and in cellular proteins (34). The structure of pRb (and probably of other members of the family) is altered by phosphorylation events, changing its binding affinities (35, 36). Despite the well conserved pocket protein domain, p107 and p130 are closer to each other than either is to pRb (37). They share a motive in the spacer region, not present in pRb, which binds cyclin A-CDK2 and cyclin E-CDK2 complexes. Moreover, they also share a sequence, absent in pRb, next to the N-terminal region (38–40).

## The Functions of Rb Proteins

The most relevant function of *Rb* family is to control cell cycle progression (34). This role, which depends on phosphorylation, is determined by the interaction with other proteins, including different transcription factors and nuclear matrix domains. Phosphorylation is carried out by cyclin-dependent kinases (41) which, in turn, are regulated by cyclins and cyclin-dependent kinase inhibitors (42). Hypophosphorylated *Rb* family members bind to distinct E2F transcription factor family members, thus promoting their inhibition. Moreover, the pocket protein-E2F complexes can act as an active mechanism for transcriptional repression (43, 44). Sequential phosphorylation of the pocket proteins leads to E2F release, allowing gene expression and cell cycle progression (43, 44).

E2Fs transcription factors form a superfamily consisting of E2F and DP (dimerization partner) proteins (45). DP family proteins are cofactors of E2F factors and DP-E2F dimerization increases DNA affinity and gets efficient regulation of the transcription. There are 8 E2F gene members that produce 10 different proteins (E2F3 and E2F7 have two isoforms). They display different affinity to pocket proteins. pRb binds principally E2F1–4; p107 binds E2F4 and 5, mostly in cycling cells, whereas p130 preferentially binds E2F5. E2F family members have unique and redundant functions in cell cycle regulation and also show cell cycle-independent roles (46). Functionally, E2Fs are divided in activators (E2F1–E2F3a) and repressors (E2F3b–8) based on structurally and affinity differences. E2F1–E2F6 factors have a domain to bind DP and DNA, E2F1–5 have a pocket protein binding domain, and E2F7 and 8 can bind DNA without DP proteins. E2Fs have transactivation and repression domains. When retinoblastoma proteins are bound to E2F factors mediate transcriptional repression.

During cell cycle, the three *Rb* family members can interact with E2Fs in a distinct, specific fashion. Activator E2Fs associate exclusively with pRb, whereas p107 or p130 bind mainly to repressors E2Fs. Importantly, pocket proteins are differentially expressed along the cell cycle, probably reflecting specific functions for each protein during the different cell cycle phases. In general, pRb and p107 are expressed in cycling cells, while p130 is preferentially expressed in quiescent cells, but this aspect is also cell type specific (43, 47, 48).

Besides their cell cycle functions, pocket proteins are involved in other cellular process such as differentiation, apoptosis, angiogenesis, or senescence modulating gene transcription (49–53). Some of these functions are also mediated by interaction with specific E2F family members (54, 55). However, they can do it both directly and indirectly, and in a very broad scale, recruiting co-repressors/activators to specific transcription factors. Recent studies propose Rb proteins to bind co-repressors: histones deacetylases (HDAC1, HDAC2) (56–58), histone de-methylases (RBP2) (59), DNA methyl transferases (DNMT1) (60), helicases (Brg1, Brm) (61, 62), histone methyl transferases (Suv39h1, RIZ, suv4-20h1/h2) (63–65), and histone binding proteins, like HP1, regulating chromatin structure and transcription (63, 66). In this context, the interaction with E2F directs pocket proteins to DNA domains leads to transcriptional repression. Finally, a recent work suggests a role for *Rb* family in influencing the chromatin structure of larger genomic regions, and also in genome stability maintenance (67).

## Pocket Proteins and Cancer

*Rb* family functions in multiple processes may exemplify their role as potential tumor suppressors. Most, if not all, of the so called hallmarks of cancer (68, 69) are regulated by this family. Accordingly, the Rb/E2F pathway is disrupted in probably all tumors (34). This subversion occurs mainly by overexpression or mutation of cyclin-dependent kinases, inactivation of CKIs, increased expression of cyclins and, in some cases, amplification and increased expression of specific E2F members. Deletion and inactivating mutations of pRb are restricted to very few specific cancer types, whereas alterations of p107 and/or p130 in human cancers is still a matter of debate, being rarely mutated in human tumors (70, 71), probably due to pRb controls E2F activity in a broad manner (72). The reason why different tumors preferentially display one or another alteration of the *Rb* pathway is still unknown. However, the most frequent alterations tend to the functional inactivation of the three *Rb* family members, indicating their potential overlapping function as tumor suppressors. In spite of this, due to the specific functions of pRb in cell differentiation and senescence, it appears to exert specific tumor suppressor activities over p107 and p130. This has been highlighted by the findings in GEMMs (Table 1).

**Table 1:** **Phenotypic abnormalities observed in germline-ablated mouse models affecting retinoblastoma family members**.

Genotype	Strategy	Lethality	Phenotype	Reference
Rb^−/−^	Germline	Embryonic (13.5)	Developmental defects, increased apoptosis	(73–75)
Rb^+/−^	Germline	Viable	Intermediate lobe pituitary tumors-cell adenomas, c-cell hyperplasia in the thyroid gland	(74, 76–78)
Rb^+/−^; p107^−/−^	Germline	Viable	Growth retardation, increased mortality rate during the first 3 weeks after birth. Multiple retina dysplastic lesions. No more tumor prone when compared with Rb^+/−^mice	(79)
Rb^−/−^; p107^−/−^	Germline	Embryonic (11.5)	Accelerated apoptosis in the liver and the central nervous system	(79)
p107^−/−^; p130^−/−^	Germline	Birth	Deregulated chondrocyte growth, defective endochondral bone development, shortened limbs, and neonatal lethality. Impaired terminal differentiation. Decreased number of follicles, developmental delay in hair, whiskers, and tooth germs	(80, 81)
E2F1^−/−^	Germline	Viable	Tissue-specific tumor induction, tissue atrophy	(82–84)
E2F2^−/−^	Germline	Viable	T lymphocyte homeostasis defects leading to a lupus-like autoimmune disorder. Negative regulator of the immune response suppressing cellular proliferation of activated lymphocytes	(85)
E2F3^−/−^	Germline C57BL/6	Neonatal	Congestive heart failure. No tumor development	(86, 87)
E2F3^−/−^	Germline 129/sv	Embryonic	Proliferation defects	(86, 87)
E2F1^−/−^; E2F2^−/−^	Germline	Viable	Limited lifespan, polyuria, polydipsia, and appeared lethargic prior to death	(85, 88)
E2F1^−/−^; E2F3^−/−^	Germline	Embryonic	Impaired development. Overlapping roles in development/maintenance of several tissues	(87, 88)
E2F2^−/−^; E2F3^−/−^	Germline	Embryonic	Central role of E2F3 in mouse development	(88)
Rb^+/−^; E2F3^−/−^	Germline		Smaller pituitary tumors. Novel tumorigenic lesions	
Rb^+/−^; E2F1^−/−^	Germline	Viable	Increase lifespan of Rb^+/−^. Reduce frequency of pituitary and thyroid tumors	(84)
Rb^+/−^; E2F3^+/−^	Germline 129/Sv	Viable	Little increase lifespan of Rb^+/−^. Smaller pituitary tumors; E2F3 acts to promote the development of tumors in Rb mutant mice	(89)
Rb^+/−^; E2F3^+/−^	Germline mixed 129/SvXC57BL/6	Weaning	Increase lifespan of Rb^+/−^	(89)
E2F3a^−/−^	Germline	Viable	No detectable effects. Low penetrance proliferation defect *in vitro*	(90)
E2F3b^−/−^	Germline	Viable	No detectable effects	(90)
E2F3a^−/−^; E2F1^−/−^	Germline	Birth (neonatal)	Cartilage defects, proliferation defects *in vitro*; overlapping functions of E2F3a and E2F1. E2F3a could substitute E2F1 and E2F3 in most murine tissues	(90)
DP1^−/−^	Chimera	Embryonic	Failure of extra-embryonic development	(91)
Rb^−/−^	Chimera	Viable	Die 3–11 months of age due to pituitary gland tumors; no retinoblastoma tumor development	(92)
Rb^+/−^; p107^−/−^	Chimera	Viable	Wide spectrum of tumors (pituitary gland, cecum, bone, lymphoid tissue). No retinoblastoma development but retinal dysplasia	(93)
Rb^−/−^; p107^−/−^	Chimera	Viable	Development of retinoblastoma at early age	(93)
Rb^+/−^; p130^−/−^	Chimera	Viable	Thymoma, hepatoma, Leydig cell tumor, insulinoma, and adrenal gland tumor. None of the tumors were seen in more than one animal	(93)
Rb^−/−^; p130^−/−^	Chimera	Viable	Retinoblastoma, pheochromocytoma, and hyperplasia of neuro-endocrine epithelial cells of the bronchus. Early death	(93)
Rb^−/−^; E2F3^−/−^	Chimera	Viable	Suppresses the formation of cataracts while aggravating the retinal dysplasia; dispensable for the development of pRb-deficient pituitary and thyroid tumors; suppresses the pulmonary neuro-endocrine hyperplasia of Rb^−/−^chimeric mice	(94)
Rb^−/−^; E2F4^−/−^	Chimera	Viable	Reduce incidence of pituitary tumors. Delay development of tumors	(95)

## Germline Knockouts of Rb Family Members

Retinoblastoma was the first tumor suppressor gene eliminated by targeted deletion in mice (73–75). Rb-null mice die during embryonic development due to multiple embryonary and extraembryonary tissue defects. However, their progress until late stages of development, indicating that pRb is not essential during early normal mouse development. This aspect was particularly emphasized by the generation of chimeric mice with a very high component of *Rb*-null cells (Table 1 and references therein).

Nevertheless, the early lethality precludes the analysis of the *Rb1* gene roles in adult mice. This is in contrast with the targeted deletion of any of the other pocket protein family members, as p107 or p130 deficient mice do not show any obvious phenotype and no tumor predisposition has been observed in any mutant animal (79, 80). In contrast, mice having simultaneous inactivation of p130 and p107 genes show neonatal lethality and limb development defects (80). These findings demonstrate the existence of compensatory roles among the pocket protein family members (i.e., p130 fulfills p107 functions in its absence or *vice versa*) and also that, in certain tissues and cellular settings, p107 and p130 perform shared growth-regulatory functions that are not fulfilled by pRb. In agreement, the embryonic lethality of Rb-null mice occurs earlier when is accompanied by the deletion of any other pocket protein (79, 96).

Genetically deficient cells for all the three *Rb* family members (TKO mouse embryonic fibroblasts) are resistant to G_1_ arrest (97, 98). Chimeric embryos composed of TKO cells develop until day 9 of gestation and some cells are able to arrest in G_0_/G_1_, to exit cell cycle and to differentiate (in teratomas and in culture), pointing to a cell type dependent mechanism and illustrating the robustness of cell cycle regulatory networks (99). Regarding the role of *Rb* family in cancer, KO mice have also provided important clues. To circumvent the embryonic lethality due to *Rb1* deficiency, heterozygous mice have been widely employed. Different studies gave evidences of compensatory roles between retinoblastoma family members (see Table 1). Lee and colleagues provided *in vivo* evidence that p107 and pRb have overlapping functions in development and adult tissues of mutant mice for the first time (79). pRb and p107 interact with E2Fs in a different manner, which could explain specific and related functions (100). As the *Rb*^+/−^ and *Rb*^−/−^ chimeric mice do not develop retinoblastoma, additional mutations besides loss of pRb function are needed to induce this type of tumor. p107 plays a tumor suppressor function in absence of pRb (101), and is responsible for a limited tumor spectrum observed in mice which have lost pRb in a variety of tissues (93). In *Rb*^−/−^ mice, the lack of *E2F4* suppresses pituitary and thyroid tumors formation, with enhancement of p107 and p130 levels associated with the activator E2Fs. This may indicate that Retinoblastoma family acts as a tumor suppressor possibly regulating activator E2Fs rather than establishing repressive E2F complex. Accordingly, in those tissues with very low p107 and p130 expression levels, pRb compensation is unlikely, making those tissues more susceptible to tumor development as a consequence of *Rb1* loss (102). In addition, the different phenotypes observed could be related to the diverse capacity of the Rb family members to interact with target proteins (103).

## Rb Family and Skin

Although numerous indirect evidences have suggested a role of pRb pathway in epidermal homeostasis (104–108) and in skin carcinogenesis (109–115), the early lethality of *Rb*-null mice precluded the study of actual *Rb1* functions in skin. Nonetheless, conventional knockout models allowed us to demonstrate that the simultaneous absence of p107 and p130 produces severe skin abnormalities affecting terminal differentiation and the expression of several morphogens involved in the inductive signals between epithelium and underlying mesenchyme (81, 116). Importantly, these signals are essential in epidermal stem cell homeostasis and have been also involved in epidermal tumor development (13).

Long-lived adult stem cells have more chances to accumulate the number of genetic hits essential for tumor development (13), and many pathways implicated in stem cell quiescence are deregulated in tumor progression. Remarkably, *Rb* family activity also plays a critical role to balance proliferation and cell survival in human embryonic stem cells (117). In epidermis, tissue homeostasis is strictly dependent on the functionality of epidermal stem cells. Whole transcriptome analysis and bioinformatic approaches using these purified epidermal stem cells revealed that the Rb-E2F axis is also an essential mediator of stem cell quiescence (118). Moreover, transgenic mouse models showed that the aberrant accumulation of altered stem cells in hair follicles and their subsequent migration to the interfollicular epidermis contribute to HPV-induced tumor development (17), which is also mediated by *Rb* family inactivation (24).

The development of an epidermal-specific *Rb*-deficient mouse model and its combination with several other GEMMs has allowed us to establish a comprehensive framework for a better understanding of the functions of the Rb-dependent signaling in different aspects of epidermal homeostasis, carcinogenesis, and metastatic development (Table 2).

**Table 2:** **Phenotypic Skin abnormalities observed in mouse models lacking pRb in epidermis**.

Genotype	Strategy	Lethality	Phenotype	Reference
Rb^f/f^; K14cre	Conditional	Viable	Enhanced proliferation, abnormal differentiation. No spontaneous tumor development	(119)
Rb^f/f^; K14cre; p130^−/−^	Conditional	Viable	No differences with Rb^f/f^; K14cre mice. Altered genomic profile	(120)
Rb^f/f^; K14cre; p107^−/−^	Conditional	Postnatal day 10	Transplants of newborn skin lead to spontaneous tumors development	(121, 122)
Rb^f/f^; K14cre; p53^f/f^	Conditional	Viable	Develop spontaneous squamous cell carcinoma, accelerated respect p53-deficient mice in epidermis	(123)
Rb^f/f^; K14cre; p21^−/−^	Conditional	High mortality rate	Hyperplasia, hyperkeratosis, inflammatory infiltrates (pnd 30). Phenotype aggravated compared with *Rb1-* or p21-deficient mice. Spontaneous epithelial tumors, preferentially in tongue and oral tissues	(124)
Rb^f/f^; K14cre; Pten^f/f^	Conditional	Increase mortality at postnatal life	Transplants of newborn skin lead to the development of spontaneous tumors. All die by 2 months of age	(125)
Rb^f/f^; K14creER^TM^	Inducible	Viable	Enhanced proliferation, abnormal differentiation. No spontaneous tumor development	(125)
Rb^f/f^; K14creER^TM^; p107^−/−^	Inducible	Viable	Aggravated phenotype of Rb^f/f^; K14cre mice: impair terminal differentiation and abnormal proliferation. Spontaneous squamous cell carcinomas in oral areas. Lifespan 5–6 months due to animal fragility	(125)
Rb^f/f^; K14creER^TM^; E2F1^−/−^	Inducible	Viable	Impair terminal differentiation and abnormal proliferation. Spontaneous squamous cell carcinomas with high penetrance	(126)

We and others demonstrated that pRb absence in epidermis is characterized by moderate hyperplasia and hyperkeratosis associated with increased proliferation and altered differentiation (121, 127). Although these characteristics might suggest a cancer-prone phenotype, no spontaneous tumor development was observed even after a long latency (121, 127). This might indicate the possible overlapping roles of the other retinoblastoma family members, p107 and p130, in suppressing skin tumorigenesis in the absence of pRb. In support of this, the phenotypic changes observed in pRb-deficient skin are aggravated by concomitant HPV E7 oncogene expression (127), indicating the involvement of other E7 targets (such as p107 and/or p130). Importantly, similar findings suggesting this compensatory or/and overlapping mechanisms have been also suggested for multiple tissues including retina, mammary gland, muscle, bone, etc. (128).

Loss of p130 did not further aggravate the phenotypic consequences of pRb ablation in skin, suggesting a potential absence of functional compensation between these two proteins in skin (120). In agreement, multiple cell cycle and proliferation genes showed a similar pattern between keratinocytes lacking pRb alone or both pRb and p130 (120). In spite of this, gene profiling analyses demonstrated that the combined absence of pRb and p130 generated changes in a large number of genes compared to pRb, indicating a primary role of p130 in modulating transcription through the specific interaction with particular E2F proteins in the absence of pRb (120). Further studies also showed a potential link between p130, specific E2Fs and chromatin remodeling machinery through the cyclin-cdk inhibitor p27 (129).

In contrast with the moderate epidermal phenotype produced by the absence of pRb or combined pRb and p130 epidermal loss, the lack of p107 and pRb in epidermis produced a severe phenotype consisting of dramatic growth retardation, no hair, and death by postnatal day 10 (121). Importantly, whilst the phenotype was aggravated with the progressive loss of one or both alleles of p107, a single functional copy of pRb was sufficient to rescue all epidermal defects (121). These data demonstrated a functional overlap between pRb and p107 in epidermis and illustrated a dose dependent effect of p107 *in vivo* in the context of *Rb1* deficiency, suggestive of a potential tumor suppressor role for p107 in the absence of pRb. This was further supported by the findings obtained with skin grafts (used to circumvent the perinatal lethality), which invariably evolved to differentiated SCC (122). Furthermore, primary double deficient keratinocytes displayed a high sensitivity to Ha Ras-mediated transformation and a particular resistance to oncogene-induced senescence (122). Importantly, biochemical and whole transcriptome analysis suggested a possible impaired p53 function in these double deficient keratinocytes (122).

Two-stage chemical carcinogenesis protocols in epidermal-specific *Rb*-deficient mice showed that the absence of pRb lead to the generation of fewer and smaller tumors than in control animals (119), but with increased malignant conversion to SCCs. Biochemical analyses indicated that, in the absence of pRb, multiple pathways, including the aberrant p53 induction mediated by E2F/p19ARF, are activated, leading to increased tumor apoptosis (119). Importantly, the increased expression and activity of p53 generates a selective pressure leading to premature p53 loss and then increased malignant conversion (119, 130). This hypothesis was also reinforced by the observation of restored susceptibility to tumor development obtained in mice lacking pRb and a single p107 allele, upon similar carcinogenesis protocols (131).

The above commented findings pointed to a primordial role of p53 in suppressing skin tumorigenesis, as suggested previously (8, 132, 133), and also indicated a possible cooperative roles of both tumor suppressors in skin, in agreement with the findings in other tissues (76, 134–139). To demonstrate such hypothesis, we generated mice lacking both *Rb1* and *Trp53* genes in epidermis (123). We observed that the spontaneous SCC development due to epidermal loss of p53 (8) is severely accelerated in mice lacking pRb and p53 (123), whereas the epidermal proliferation and differentiation phenotype due to pRb loss is not enhanced by the simultaneous inactivation of pRb and p53 (123). Detailed analyses indicated that tumorigenesis due to *p53* loss was associated to early chromosome instability, and under these settings the increased proliferation promoted by the absence of *Rb1* resulted in an accelerated process (140). Of note, the transcriptome analysis of tumors arising in these deficient mice revealed a highly significant overlap with human tumors. These were characterized by p53 mutation, poor prognosis and/or very high predisposition to develop distant metastasis (141), regardless the tissue of origin, and including clinically relevant human cancers such as breast and lung tumors (141, 142). In agreement, squamous cell carcinomas generated in these mouse models are highly metastatic and displayed early signs of epithelial-mesenchymal transition and deregulated expression of specific miRNAs (143). The possible link between Rb family and deregulated miRNA expression has also been reported in other tissues, such as muscle and retina (144, 145), and also upon HPV E7 expression in human keratinocytes (24). Remarkably, the specific upregulation of miR-21 dependent in p53-deficient metastatic tumor cells, which appears to be mediated by increased mTOR and Stat3 activity (143), was also found in human metastatic lung tumors bearing mutated p53 gene (143), thus supporting a potential mechanism of metastatic spreading in human tumors. Nonetheless, the possible relative contribution of *Rb1* and Trp53 tumor suppressor genes to this process remains unsolved.

The cyclin-cdk inhibitor p21 is a bona fide transcriptional target of p53 (146, 147). It has been reported as responsible for the potential mechanism mediating cell cycle arrest in the absence of pRb (148–150), and it has been also involved in epidermal homeostasis and carcinogenesis (151–157). Consequently, we have recently generated mice lacking p21 in the absence of epidermal pRb. Remarkably these mice developed skin inflammatory processes followed by spontaneous tumor development, with strong resemblance to human head and neck SCCs by histopathology and transcriptome characteristics (124). Further, these mice also showed aberrant Stat3 signaling, thus reinforcing our previous findings connecting pRb, p53, and Stat3 (143).

## Acute vs. Chronic Retinoblastoma Gene Loss

Most sporadic cancers are originated by several mutations, including loss of function of tumor suppressors, occurring only in very few cells of adult tissues. To mimic such situation Sage and co-workers developed an experimental approach that allows *Rb1* elimination in an acute manner in primary mouse embryonic fibroblasts (158). This revealed a significant functional difference between such acute loss of pRb and that achieved by inactivation during embryonic development (158). Importantly, one of the main molecular bases of such difference relies on the acclimatization of the tissues to a permanent loss of pRb that allows the induction of compensatory genes, such as p107 (158). Remarkably, the proliferative arrest mediated by differentiation stimuli in keratinocytes *in vitro* also displayed similar characteristics upon acute or chronic *Rb1* loss (121). These results might have important implications for the interpretation of Rb-loss in GEMMs, and in particular in the context of epidermal tumor development.

To find out possible differences between acute and chronic *Rb1* loss in adult mice, we have recently generated a new mouse model lacking pRb in epidermis in an inducible manner by topical tamoxifen administration (125, 126). Acute pRb deletion in epidermis *in vivo* caused moderate hyperplasia due to changes in epidermal proliferation and differentiation similar to those observed after chronic pRb loss (116). This phenotype is durable along mice lifespan (125), indicative of efficient targeting in adult stem cells. However, no tumor development was observed even 1 year after recombination induction (125). These observations indicated that, although the acute loss of pRb is sufficient to overcome some processes associated to prolonged pRb ablation, the extended time required for the development of spontaneous tumors allows the induction of compensatory genes and thus tumor suppression.

In order to analyze whether p107 is responsible for this tumor suppression, we generated a mouse model susceptible of acute pRb loss in the absence of p107 (125). These mice displayed abnormal epidermal differentiation, hair loss, frail appearance, and lesions in the cheek, neck, eyelids, and snout, leading to lifespan reduction up to 6 months after recombination induction. Moreover, all mice developed spontaneous tumors starting from 2 months after recombination and affecting preferentially perioral areas (125). Biochemical and genomic analyses of these tumors and primary keratinocytes demonstrated that combined pRb and p107 absence limits the transcriptional tumor suppressive functions of p53, leading to a reduced *Pten* gene expression. Consequently, this mouse model confirmed the existence of an *in vivo* novel functional connection between the three major suppressor genes *TP53, Pten*, and *Rb1*, previously suggested from *in vitro* experiments (159). Similarly, Lambert and colleagues have reported that loss of pRb and p107 can predispose to oral tumors in mice after chemical carcinogenesis (160). However, the inactivation of all three family members was unable to induce cervical cancer (161). These findings support a tissue context role for the Rb family in tumor suppression in stratified epithelia, and the different results observed in distinct mouse strains may also point to possible genetic and/or epigenetic factors affecting their tumor suppressor functions.

The loss of pRb is accompanied by increased E2F activity and deregulated expression of several E2F members, including increased E2F1 expression (116). *E2F1* is the best described E2F family member. Although initially considered as a potential oncogene due to its ability to drive S-phase progression in quiescent cells (162, 163), data obtained in E2F1-null mice (82, 83) revealed a potential tumor suppressor role associated with impaired apoptosis induction (82, 83). Such apoptosis takes place through p53-dependent and -independent mechanisms (119, 164, 165). Such functional difference is also emphasized in epidermal carcinogenesis. E2F1 overexpression in transgenic mouse epidermis leads to spontaneous tumor development, which is accelerated by p53 loss (166, 167). However, such transgenic expression of E2F1 was found to inhibit *ras*-driven skin carcinogenesis in part through a p53/p19^arf^ dependent process (114, 168, 169). This process is also reminiscent to that observed upon chemical carcinogenesis protocols in mice lacking epidermal pRb (119). Importantly, loss of *E2F1* reduces tumorigenesis and extends the lifespan of *Rb1* heterozygous mice indicating that an important part of the several tumor suppressor activities of pRb depend on its ability to repress E2F1 (170, 171). Of note, E2F1 is dispensable for the normal skin development and homeostasis, but plays important roles during wound healing *in vivo* (107). As the elimination of *Rb1* in epidermis leads to increased E2F1 expression and activity (116), which also partially explain the abnormal susceptibility to chemical carcinogenesis protocols (119), we obtained a mouse model of epidermal inducible *Rb1* loss in an *E2F1*-deficient background (126). The epidermis of mice lacking *Rb1* and *E2F1* was characterized by generalized hyperplasia and abnormal differentiation similar to that observed in mice under acute or chronic epidermal *Rb1* loss (126). This suggests that some of the functions of *Rb1* in epidermis are E2F1-independent. Nonetheless, the combined deficiency in *Rb1* and *E2F1* results in spontaneous epidermal carcinomas with high penetrance (126). These tumors displayed a hair follicle origin and a functional p19/p53 axis (126). Whole transcriptome analysis of these tumors revealed a potential involvement of Wnt signaling, as well as a significant overlap with human tumors (126). These results demonstrated, for the first time, that tumor suppressor functions of pRb *in vivo* are partially E2F1-dependent in specific tissues.

## Concluding Remarks and Future Perspective

The retinoblastoma pathway is an essential mechanism allowing proper cell cycle progression. Therefore, not surprisingly, most human tumors show aberrant disruption of this pathway rather than mutation in Rb1 *per se*. Nonetheless, it is composed by a large number of different proteins with specific and diverse functions, and the different tumor types usually show distinct type of alterations. The analysis of mouse models bearing different combinations of genetic alterations in a tissue-specific manner, and in an acute or chronic mode, will continue to provide invaluable information on the retinoblastoma pathway and its involvement in the control of different cancer hallmarks. Such studies may highlight the significant events and the molecular entities involved in initiation and progression of the disease in different tissue contexts. In skin, the findings on the retinoblastoma family proteins and the Rb-dependent pathway support this type of studies for specific squamous cancers. Nonetheless, similar or different molecular events may take place in other tissues, and it is essential to consider that tumor development may differ in mice or humans. These factors require extremely refined comparative studies to validate the models in order to identify potential biomarkers and/or molecular targets for new therapies. Nonetheless, these mouse models could provide a highly valuable tool for preclinical research.

## Conflict of Interest Statement

The authors declare that the research was conducted in the absence of any commercial or financial relationships that could be construed as a potential conflict of interest.
